# Depletion of UNK-1 affects head locomotive behaviors in *Caenorhabditis elegans*

**DOI:** 10.17912/micropub.biology.001659

**Published:** 2025-06-11

**Authors:** Lexin Gitler, Callista Yee

**Affiliations:** 1 Shanghai American School, Pudong Campus, Shanghai, China; 2 Department of Biology, Stanford University, Stanford, California, United States; 3 Howard Hughes Medical Institute, Stanford, California, United States; 4 Department of Zoology, University of British Columbia, Vancouver, British Columbia, Canada

## Abstract

During development, transcription factors execute genetic programs to define and shape the nervous system. We previously reported the transcription factor EGL-43 /Mecom regulates expression of numerous genes important for neuronal development in *C. elegans* . Here we characterized the function of one EGL-43 target, UNK-1 /Unkempt, a conserved RNA-binding protein. Using genome editing, we introduced an auxin-inducible degron tag and mNeonGreen into the *unk-1* locus. We observed cytoplasmic expression of UNK-1 in many head neurons. Depletion of UNK-1 resulted in increased head oscillations, a phenotype typically associated with defects in neurotransmitter synthesis. Our study links UNK-1 to head motor control in *C. elegans* .

**Figure 1. Characterization of UNK-1 in C. elegans:**
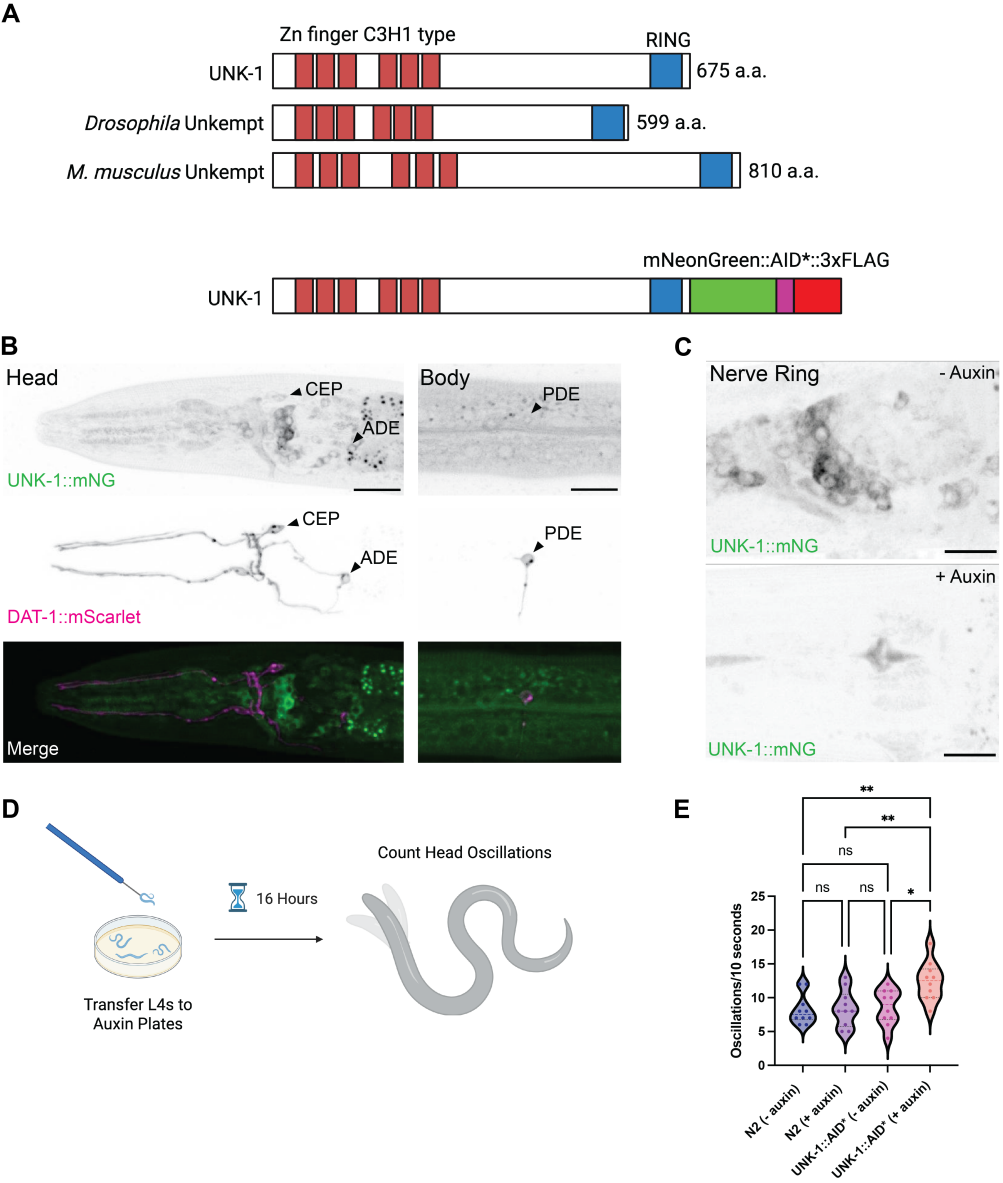
A) Top: Protein domains of Unkempt related proteins ( *C. elegans* UNK-1 , *D. melanogaster* Unkempt, *M. musculus* Unkempt); Bottom: Schematic of C-terminal fusion of UNK-1 to mNeonGreen::AID*::3xFLAG, generated using BioRender (https://biorender.com/9wf5qlq). B) Micrographs of L4 animals expressing endogenously labelled UNK-1 ::mNG (top) and DAT-1 ::mScarlet (Middle). UNK-1 is expressed in the cytoplasm of many sensory neurons, including all dopaminergic neurons (CEP and ADE in the head, PDE in the body). Scale bar for all images = 20 um.C) Micrographs of UNK-1 ::AID* day 1 adult animals expressing UNK-1 ::mNG treated with 0 mM Auxin (top) and 4 mM Auxin (bottom) overnight. Scale bar for all images = 10 um. D) UNK-1 depletion and assay schematic, generated using BioRender (https://biorender.com/qdb7g25). E) Quantification of head oscillation of day 1 adults during forward movement. Each dot represents a single animal, n = 10 for all genotypes and treatments. *p* values were calculated using ordinary one-way ANOVA with Tukey's multiple comparisons tests. ns indicates not significant, * indicates *p < 0.05,* ** indicates *p < 0.01.*

## Description

Transcription factors (TFs) define multiple aspects of neuronal identity and function. Our laboratory recently discovered that the TF EGL-43 is critical for synapse formation in dopaminergic neurons (Yee et al., 2024). EGL-43 ChIP-seq revealed that EGL-43 binds to promoters of many genes with a variety of cellular functions (Deng et al., 2020). Depletion of EGL-43 decreased levels of presynaptic protein expression (Yee et al., 2024). Likewise, directly mutating the EGL-43 binding sites within these promoters reduced corresponding protein expression. Many of the EGL-43 target genes encode proteins with unknown functions. We sought to define the function of some of these genes. We used two criteria to select genes for further study. First, we looked for genes that were evolutionarily conserved. Second, we selected genes whose functions have not been characterized in *C. elegans* . One candidate we focused on was a gene related to *unkempt* .

Originally identified in *Drosophila* (Mohler et al., 1992), *unkempt* is highly expressed embryonically and is essential for early development as homozygous deletion causes lethality during mid-pupal development. Flies carrying a hypomorphic allele of *unkempt* survive into adulthood but exhibit an “unkempt” phenotype, characterized by roughened eyes, irregular scutellar bristles, and splayed wings (Mohler et al., 1992). In mice, the *Unkempt* gene encodes a CCCH-type zinc finger domain containing RNA-binding protein that regulates RNA stability, localization, and translation, and plays important roles in development and function of the nervous system (Murn et al., 2016; Murn et al., 2015). Intriguingly, knockout of *Unkempt* in the mouse nervous system results in improved memory formation and cognitive flexibility (Vinsland et al., 2021). Little is known about the function of Unkempt in *C. elegans* . Given the well characterized nervous system of *C. elegans* , we sought to study Unkempt's function in this model system.

*C. elegans* has a single Unkempt ortholog called UNK-1 (UNKempt-related zinc finger protein). According to WormBase release WS296, the *unk-1* locus encodes 4 unique transcripts resulting in isoforms with a shared C-terminus. To study the expression and function of all isoforms of UNK-1 , we used CRISPR/Cas9 to generate a C-terminal fusion of mNeonGreen::AID*::3xFLAG to UNK-1 ( **Figure 1A** ). The mNeonGreen tag allowed us to determine where UNK-1 is expressed and its subcellular localization. The AID system was discovered in plants but adapted for use in *C. elegans* (Zhang et al., 2015). AID allows for rapid (and reversible) depletion of fusion proteins via expression of TIR1 protein, which targets proteins to the proteasome. The truncated AID* degron tag (Morawska and Ulrich, 2013) enabled us to rapidly degrade UNK-1 to study its loss-of-function phenotype. Finally, the 3x FLAG tag will allow future biochemical analyses of UNK-1 .

We isolated a single allele with the correct insertion, *unk-1 ( wy2110 ).* Following 3 outcrosses, we crossed in a ubiquitously expressed TIR1 transgene and an endogenous mScarlet-tagged allele of *dat-1* , as a marker of dopaminergic neurons. This resulting strain, TV30205 [ *wrdSi22 ; dat-1 ( wy2061 ); unk-1 ( wy2110 )]* , is referred to as UNK-1 ::AID* throughout the text. In L4 animals UNK-1 was observed in the cytoplasm of pharyngeal cells and many head neurons ( **Figure 1B** ), including weak expression in all dopaminergic neurons (CEP, ADE and PDE). This expression pattern is consistent with the known expression pattern of EGL-43 (Yee et al., 2024) and the *unk-1* expression data from the CeNGEN neuronal gene expression database (Taylor et al., 2021).

To deplete UNK-1 from worms, we treated wildtype N2 or UNK-1 ::AID* L4 animals for 16h with either 0 mM or 4 mM K-NAA (auxin). Following auxin treatment, UNK-1 expression in UNK-1 ::AID* animals was undetectable in the nerve ring of day 1 adults ( **Figure 1C** ). To broadly characterize the effect of loss of UNK-1 , we recorded short videos of worm behavior of day 1 adults following auxin treatment ( **Figure 1D** ). Auxin treatment did not lead to obvious changes in forward or backward locomotion of either genotype (not quantified). To our surprise, we observed that auxin treatment of UNK-1 ::AID* animals, but not N2 animals, led to a marked increase in head oscillations during forward movement ( **Figure 1E** ). Unfortunately due to time limitations, we were unable to assay animals carrying exclusively TIR1 or *unk-1 ( wy2110 )* .

Head oscillations are rhythmic movements that play a key role in the worm's locomotion and environmental exploration. Acetylcholine, GABA, and tyramine are three neurotransmitters that regulate normal head oscillations (Alkema et al., 2005). In some contexts, it is advantageous for the worm to suppress head oscillations. For example, when it encounters certain fungi in the wild, uncontrolled head oscillations can trigger fungus to constrict around the worm's head, entrapping it and leading to death by strangulation (Maguire et al., 2011). The ability to suppress head oscillations allows the worm to smoothly back out of the fungal trap and avoid capture. Since degradation of UNK-1 increased head oscillations, our data suggests UNK-1 is normally required to suppress this behavior.

Our study reveals an unexpected role of UNK-1 in regulating head oscillations in *C. elegans* . Since UNK-1 is an RNA-binding protein, future studies characterizing the RNAs it binds will be critical in furthering our understanding of UNK-1 function. Given the similarity between the failure to suppress head oscillations in mutants that cannot synthesize tyramine, e.g. *tdc-1* (Alkema et al., 2005), UNK-1 may function in this pathway, possibly by stabilizing *tdc-1* transcript or facilitating its translation. Future studies will involve characterization of other genetic alleles of *unk-1* and performing a developmental time course of UNK-1 depletion.

Mutants that are defective in dopaminergic neuron identity or function have not been described to possess defects in head oscillations. Although we originally identified *unk-1* as a downstream target of EGL-43 , which is critical for dopaminergic neuron function, it is unclear if dopaminergic neurons contribute to this behavior. The RMD, SMD, RME, and RIM motor neurons innervate the *C. elegans* head muscles and have been implicated in regulating head oscillations (Alkema et al., 2005). According to the CeNGEN database *unk-1* is expressed in many head neurons, including the aforementioned motor neurons (Taylor et al., 2021). It will be important to thoroughly characterize the identity of each neuron expressing UNK-1 through co-localization experiments using strains such as NeuroPAL (Yemini et al., 2021). Following this, we will use our new allele of *unk-1* to perform cell-specific degradation, as opposed to global degradation, to determine which neurons UNK-1 is required in to regulate normal head oscillations.

Evidence from *Drosophila* connects Unkempt to the mTOR signaling pathway (Avet-Rochex et al., 2014; Maierbrugger et al., 2020). It will be interesting to test if mTOR signaling converges on UNK-1 and if this connection is important for the head oscillation behavior. In summary, we have characterized the expression and function of *unk-1* in the *C. elegans* nervous system.

## Methods

**General Maintenance**

Worms were cultured on standard NGM plates seeded with OP50 as previously described (Brenner, 1974). *unk-1 ( wy2110 )* animals were outcrossed to 3x to N2 before they were used for experiments. Animals used for experimentation were reared at 20C and were well fed for at least 3 generations.

**Genome editing of *unk-1* using CRISPR/Cas9**

Guides targeting *unk-1* were designed using the IDT Alt-R™ CRISPR-Cas9 guide RNA design tool. CRISPR injection mixes were prepared as previously described (Yee et al., 2024). Young adult N2 worms were microinjected using pRF4 ( *rol-6* ) as a co-injection marker, and F1 roller progeny were singled on individual plates. F2 progeny were screened for expression of mNeonGreen and a single correct isolate was identified by Sanger sequencing. This strain, TV30179 , will be deposited into the CGC.

**Auxin-induced degradation of UNK-1**

To prepare auxin plates, molten NGM media was supplemented with K-NAA (PhytoTech Labs) to a final concentration of 4 mM. OP50 bacteria was seeded on auxin containing plates at least 2 days in advance of each experiment. L4 animals were placed on seeded auxin plates overnight (16 hours, 20C) before being subjected to microscopy or behavioral assays.

**Microscopy**

To image UNK-1 expression, animals were mounted on 2% agarose pads and immobilized using 10 mM levamisole. Animals were imaged using a spinning-disk system (3i) equipped with a CSU-W1 spinning-disk head (Yokogawa), 488-nm and 561-nm solid-state lasers, a C-Apochromat 40× 0.9 NA water-immersion objective, and a Prime95B camera (Photometrics).

**Head Oscillation Assays**

L4 animals that were treated with 0 mM or 4 mM auxin were moved onto regular seeded NGM plates and allowed to equilibrate for 15 minutes at 20C. Animals were then recorded for 30s using an iPhone 15 Pro mounted to a Zeiss SV6 dissecting microscope. Videos were processed using Apple QuickTime Player (v.10.5) and randomly trimmed into a 10s clip. Head oscillations, defined as a complete cycle of left-to-right movement, during forward crawling were counted from each clip by playing videos at 0.25 speed. The identity of the genotypes that were tested were blinded to the experimenter. Assays were reproducible and repeated three times.

**Statistical analysis and Graphing**

All statistical analysis was performed using GraphPad Prism (version 10), using ordinary one-way ANOVA with Tukey's multiple comparison tests, comparing the mean of each column with the mean of every other column.

## Reagents

N2 : Wild Type

JDW224 : wrdSi22 [eft-3p::TIR1::F2A::mTagBFP2::AID*::NLS:: tbb-2 3'UTR] I

TV30179 : *unk-1 ( wy2110* [ unk-1 ::mNeonGreen::AID*::3xFLAG ]) X

TV30205 : wrdSi22 [eft-3p::TIR1::F2A::mTagBFP2::AID*::NLS:: tbb-2 3'UTR] I; dat-1 ( wy2061 [ dat-1 ::mScarlet]) III; unk-1 ( wy2110 [ unk-1 ::mNeonGreen::AID*::3xFLAG]) X
